# COVID-19 Vaccine Hesitancy among Young Adults in Saudi Arabia: A Cross-Sectional Web-Based Study

**DOI:** 10.3390/vaccines9040330

**Published:** 2021-04-01

**Authors:** Dalia Almaghaslah, Abdulrhman Alsayari, Geetha Kandasamy, Rajalakshimi Vasudevan

**Affiliations:** 1 Department of Clinical Pharmacy, College of Pharmacy, King Khalid University, P.O. Box 1882, Abha 61441, Saudi Arabia; glakshmi@kku.edu.sa; 2 Department of Pharmacognosy, College of Pharmacy, King Khalid University, P.O. Box 1882, Abha 61441, Saudi Arabia; 3 Department of Pharmacology, College of Pharmacy, King Khalid University, P.O. Box 1882, Abha 61441, Saudi Arabia; raja@kku.edu.sa

**Keywords:** vaccination hesitancy, Saudi Arbia, COVID-19, vaccine reluctance

## Abstract

Ending the COVID-19 pandemic requires achieving herd immunity, either by previous infection or by vaccination. However, concerns about the COVID-19 vaccine are growing around the globe. The current study was conducted to investigate young the adult population’s hesitancy towards the vaccine. The study used a prospective cross-sectional design. Data was collected using an online self-administered questionnaire. A total of 862 Saudi adults participated. Information was gathered on the participants’ perspectives towards the severity and susceptibility of the COVID-19 infection, reasons for their hesitancy to receive the vaccine, perceived benefits, and reasons for action. Just under a quarter (19.6%) of respondents had previously tested positive for COVID-19. A small minority of the participants had already received the vaccine (2.1%), while 20.3% had registered in the Sehaty app (application) to receive the vaccine. Just under half of them (48%) will take the vaccine when mass vaccination is achieved and approximately the same number (46.7%) will only take it if it is made mandatory. Vaccine reluctance is highly prevalent among the general public in Saudi Arabia during the COVID-19 pandemic. While many are aware of a high likelihood of getting the infection, the efficacy and safety of the COVID-19 vaccine were reported as barriers to vaccination.

## 1. Introduction

The 2019 coronavirus disease (COVID-19) outbreak was declared to be a pandemic by the World Health Organisation (WHO) after the global spread of the virus on the 1st of March 2020 [1,2].

In the spring of 2020, governments worldwide implemented precautionary measures, such as social distancing, quarantine, and mask-wearing, to control the spread of the disease and its related death tolls [3]. Despite the preventative strategies, European countries were hit by a second wave of infection in the autumn of 2021 that required them to tighten the preventative measures that were eased during summer, 2020 [3]. Saudi Arabia adopted the same preventative efforts as the rest of the world [1]. However, this country, as well as the neighbouring Gulf countries, were hit by a second wave the beginning of February 2021, necessitating the reinforcement of the previous curfew. These intermittent strategies are expected to continue to operate until herd immunity, or population immunity, is developed either by vaccination or previous infection [4,5]. Considering that social distancing is limiting COVID-19 transmission to a minimum, achievement of herd immunity can only be reached through mass vaccination [3,4]. 

According to the WHO, three COVID-19 vaccines have been approved by the national regulatory authorities [5]. The Centers for Disease Control and Prevention (CDC) and the Food and Drug Administration (FDA) listed two approved vaccines: Pfizer-BioNTech COVID-19 Vaccine and Moderna COVID-19 Vaccine [6,7]. However, more than 53 vaccines are currently in clinical trials on humans [8]. 

In Saudi Arabia, the Ministry of Health launched a vaccine campaign through a mobile application named Sehaty, which facilitates registration for COVID-19 vaccination. Vaccination centres were also established in different cities around the country (Ministry of Health (MOH), no date). The campaign was launched on the 17 December 2020, offering the Pfizer-BioNTech COVID-19 Vaccine, and aims to provide free vaccination to all citizens and residents [9]. The Ministry of Health and the Saudi Centre of Disease Prevention and Control identified a target population for each phase of the roll-out. The first phase targeted people who are over the age of 65 years, as well as those working in occupations carrying the most risk of infection (obese people i.e., Body Mass Index > 40, Immunocompromised patients, and patients with two or more chronic conditions). The second phase targeted people who are above the age of 50 years, healthcare professionals, those who have one chronic condition, or people with a BMI between 30–40. The third phase will be open to all the public who wish to receive the vaccine [9].

Several studies have been conducted to assess the public views on the COVID-19 vaccination, as well as vaccine hesitancy [10]. Concern about vaccine hesitancy is growing around the globe [11].

“Vaccine hesitancy” refers to the reluctance or unwillingness to be vaccinated or have one’s children vaccinated against a disease, even if the vaccine is proven safe and effective [12]. A global survey assessing the acceptance rate of the COVID-19 vaccine in 19 countries found that (71.5%) of participants stated that they would be very or somewhat likely to receive a COVID-19 vaccine, while 48.1% stated that they would accept their employer’s suggestions to do so [11].

A nationwide study in Indonesia showed a high level of acceptance of free vaccination with an efficacy of 95% [13]. Another nationwide study, this one in China, revealed that the majority of study participants intended to obtain the COVID-19 vaccination [4]. A survey in France indicated that vaccine acceptance is multifactorial, dependent on vaccine efficacy, national vaccination policy, as well as some demographic factors [3]. A review article, however, showed a high rate of hesitancy to COVID-19 vaccinations in certain countries, such as France and Russia [10]. The safety and efficacy of the vaccine were the top reasons for hesitancy; other reasons included lack of information regarding eligibility, worry about the possible side effects, refusing vaccination on principle, inconvenience, and lack of time [10].

In Saudi Arabia, a single study was carried out before the vaccines were approved or launched at a national level. The study showed that 67% of respondents intended to receive the hypothetical vaccine, and only (7%) were hesitant to take it [4]. 

However, the number of people who have registered to take the vaccine through Sehaty, just over 1 million, is far below the number required to develop herd immunity (60% of the population) or 20,400,000 out of 34 million of the total population of Saudi Arabia [3]. Since vaccine distribution in the country was rescheduled due to the manufacturer’s delay, the current study was conducted to investigate the adult population’s hesitancy towards the vaccine [14].

## 2. Methods

### 2.1. Study Design

This study used a web-based cross-sectional design.

### 2.2. Population and Setting

This study was conducted to assess the public’s beliefs towards the COVID-19 vaccine and the reasons for a hesitancy among the general Saudi population to receive it. The web-based data collection took place between 15 January and 7 February 2021, just after the second wave of COVID-19 pandemic hit the country.

### 2.3. Sample Size and Sampling Procedure

The sample was a self-selected non-probability sample of social media users aged ≥18 years and residing in Saudi Arabia.

The sample size was calculated using the Raosoft sample size calculator and based on the total population (34,218,169), with a 95% confidence interval; thus, the minimum sample size was set to be 385. The web-based data collection tool was designed using Google forms and was distributed via social media applications i.e., WhatsApp and Instagram. The invitation letter was sent via WhatsApp groups and was posted on some community groups on Instagram. The invitation letter explained the aim of the study and the approximate time required to complete the questionnaire. Participants were asked to further distribute the survey among their social networks. A total of 862 participants were recruited.

### 2.4. Data Collection Tool

The questionnaire consisted of three domains: demographic information, health status, and infection history. The last two sections used a Likert scale to assess participants’ perspectives towards the severity and susceptibility of COVID-19 infection, reasons for hesitancy, perceived benefits, and reasons/causes for action. The questionnaire was adapted from previous studies [15,16].

The questionnaire was prepared in the English language, then translated into the Arabic language. Language validity was undertaken by retranslating the Arabic version of the questionnaire into English, to ensure that the original meaning of the questions was preserved (back translation). The authors, who are bilingual speakers of both English and Arabic, conducted the back translation. The questionnaire was distributed in Arabic.

### 2.5. Ethics Approval

An ethical clearance was given by The Ethical Committee of the Scientific Research, King Khalid University ECM#2021-3704. All respondents were asked for their consent before participation in the study.

## 3. Results

Table 1 shows the demographic information of respondents. More than half of the participants (64.3%) were between the ages of 18–29 years. Just over half of participants (54.5%) were females and were educated to a university level and above (67.5%). A small minority of participants had chronic conditions (6%). Just under a quarter (19.6%) had previously tested positive for COVID-19. Very few participants had already received the vaccine (2.1%), while 20.3% had registered in the Sehaty app to receive it.

Table 2 shows participants’ views on the severity and likelihood of getting COVID-19 infection. More than half of the participants (55.9%) agreed or strongly agreed that COVID-19 is a serious condition and requires vaccination. A minority of participants agreed or strongly agreed that developing a natural immunity is not enough to prevent COVID-19 infection (12.4%) and that the probability of getting the infection had decreased (15.8%).

Table 3 illustrates the reasons behind their hesitancy towards getting the vaccine, its possible benefits, and causes of action. More than half of participants (53%) agreed or strongly agreed that eligibility requirements and the registration process are not the reasons behind their hesitancy. Almost half (49.9%) agreed or strongly agreed that vaccine effectiveness is among the reasons for their hesitancy. Only one third (33.1%) of participants agreed or strongly agreed that news on social media was the reason for their refusal of the vaccine, while 39% of participants agreed or strongly agreed that receiving the vaccine would make them less worried. Just under half (48%) agreed or strongly agreed that vaccination would reduce the likelihood of catching the infection and approximately the same number (46.7%) agreed or strongly agreed that mass vaccination would help relax the preventative measures currently in place.

Thirty-nine percent of participants agreed or strongly agreed that they were being provided with sufficient information about the vaccine’s efficacy and safety, which would encourage them to take the step to register for the vaccine, while just under half of them (48%) will take the vaccine when mass vaccination is achieved and approximately the same number (46.7%) will only take it if it is made mandatory.

## 4. Discussion

The COVID-19 vaccine was launched by MOH in mid-December 2020, but the number of people who registered to receive it was far below what is required to end COVID-19 at a national level i.e., by developing herd immunity through mass vaccination.

Hence, the current study was conducted to investigate the Saudi population’s perceptions towards COVID-19 vaccinations. The findings indicate that only 2.1% of the respondents have received the vaccine and only 20.4% have registered through the Sehaty app to receive it. These figures could be interpreted as a high level of hesitancy toward vaccination, especially with the vaccine being offered free of charge for all residents of the country. However, two-thirds of the respondents (64.3%) are young adults between the ages of 18–29; hence, they are not particularly vulnerable to COVID-19 complications nor are they a high-priority group to receive the vaccine at the beginning of the national vaccination campaign run by the MOH and the Saudi Center for Disease Prevention and Control.

In contrast, a previous study conducted in Saudi Arabia revealed an acceptance level of 67% [4]. The reason for this opposite finding is likely due to the fact that the previous work was carried out before any vaccine was approved, so this was a hypothetical perception rather than a true intention to take the vaccine. The current study findings are in line with an MOH report in respect to the number of people who are registered in the Sehaty app.

The United Kingdom and Ireland have reported resistance levels of 31% and 35%, respectively [17]. A U.S. poll showed that 50% of Americans intend to be vaccinated, 30% were not sure, and 20% indicated vaccine resistance. Another study in the U.S. found that the probability of Americans getting a COVID-19 immunization were: very likely (52%), somewhat likely (27%), not likely (15%), definitely not (7%) [18]. In Indonesia, the level of vaccine acceptance is very high at 93.3%, [13] while in China, just over half reported a “probably yes” intent (54.6%), followed by a “definite yes” intent (28.7%). Vaccination resistance varies widely across countries and is dependent on many factors, including socio-demographic characteristics, the efficacy and safety of the vaccine being used, having children at home, political affiliation, the perceived likelihood of getting infected with COVID-19, perceived COVID-19 severity, as well as the affordability of the vaccine.

Evaluation of the perceived severity and susceptibility of COVID-19 infection revealed that 55.9% of respondents were aware of the severity of the disease and its complications. The fact that only 19.6% had previously tested positive for COVID-19 contributed to their perception that natural immunity is enough to prevent reinfection, as a minority of respondents (12.4%) agreed or strongly agreed with that statement.

The majority of participants (63.6%) were aware that the probability of catching COVID-19 is still high, despite the decreasing numbers of reported positive cases. The media awareness campaign provided by the MOH in the form of a daily detailed report of the number of active cases, number of recovered cases, number of critical cases, and number of deaths per city since the beginning of the pandemic, had definitely raised population awareness about the severity and susceptibility of COVID-19. The government has also raised awareness through an electronic health system in the form of social distancing apps (Tawaklna) that provide health services such as Caution Mode of confirmed cases and COVID-19 test results, as well as Permit Services to be used during quarantine. Another app is Tabaud that shows exposure to COVID-19 active cases.

As stated in previous studies, the vaccine’s efficacy and safety were among the top reasons for vaccine reluctance. These are not unsubstantiated reasons, since some conflicting findings have been reported regarding the available vaccines. For example, the clinical trial by Polack et al. (2020) that assessed the efficacy and safety of Pfizer BioNTech covid-19 vaccine (BNT162b2) concluded that a two-dose regimen of the vaccine was 95% effective in preventing Covid-19 (95% credible interval, 90.3 to 97.6) in people aged 16 and older. It also reported a safety profile over a median of 2 months that is similar to other viral vaccines [19].

However, in Israel, where more than 75% of the elderly population received the first dose of the vaccine, the rollout findings reported a lower reduction in cases (33%) compared to that found in clinical trials (52%). The long-term side effects of the new mRNA vaccine are still unknown, as well as whether the vaccine will provide lasting immunity [20]. Hence, some of the public concerns regarding the long-term efficacy and safety of the vaccine are valid.

Although just one third of respondents (33.1%) agreed or strongly agreed that they were influenced by the anti-vaccination movements, the distrust of the private pharmaceutical companies and their intentions have contributed to the misinformation and public distrust of official authorities. Vaccine literacy programs should be tailored, depending on the level of health, scientific and general literacy of the high-priority groups. Strategies to tackle vaccination reluctance should be designed to eradicate community-specific anti-vaccination misconceptions [11].

The psychological impact of COVID-19 and its precautionary measures have been extensively researched [21]. The pandemic negatively affected people’s day-to-day lives and their careers, and consequently impacted their health and wellbeing. A nationwide study in Saudi Arabia reported moderate to severe depression, anxiety, and stress as a result of the pandemic among one third of respondents [22]. For that reason, it is not surprising that the perceived benefits of vaccination, including less anxiety, less likelihood of getting infected with COVID-19, and easing of the preventative efforts were encouraging factors, as noted by 39%, 48%, and 46.7% of respondents, respectively.

Among the reasons given for obtaining a vaccination, achieving mass vaccination was found to be a driving force by 48% of participants. As mentioned previously, participants had concerns about the efficacy and safety of the vaccine; hence, having a majority of the population receiving it would probably provide some reassurance regarding their concerns.

The second strongest motive for taking the vaccine was making it mandatory (46.7%). Ending the COVID-19 pandemic requires developing herd immunity, which might not be achieved through voluntary vaccination rates [23].

However, vaccine mandates would have legal and ethical considerations. Some argue that vaccination should be compulsory for certain groups, including health care workers and businesses that require in-person attendance or serving vulnerable populations. Students and faculty members, as well as schoolchildren, teachers, and staff might require vaccinations in order to facilitate their return to safe classroom education [24]. Others suggest that children are not a high priority group and suggest that high-risk groups such as active-duty military, Veterans Affairs facilities, federal prisons, and immigration detention centres receive early vaccinations [25].

On the other hand, a global survey concluded that compulsory vaccination by employers increased refusal of vaccinations across participants of different nationalities. Hence, voluntary vaccination promotions are a better option. The involvement of trusted non-government organizations and community-based groups is crucial to building trust in COVID-19 vaccination [25].

The findings in this study are subject to several limitations. The use of an online questionnaire limits the recruitment to individuals who are digitally literate, thus a majority of these participants were between the ages of 18–29 years. Hence, the opinions of digitally illiterate people were underrepresented in the current study. Also, this young age group is not particularly vulnerable to COVID-19 complications, nor are they a high-priority group to receive the vaccine in the early stages of the national vaccination campaign, so they are more likely to have vaccine hesitancy. Additionally, the data was collected while the vaccine supply was suspended due to manufacturer’s delay, so participants’ registration to take the vaccine might have been affected by this circumstance. Using social media in collecting data might have limited the variety of the study sample.

## 5. Conclusions

The research provides a snapshot into COVID-19 vaccination hesitancy among Saudi adults. It revealed that vaccine reluctance is highly prevalent among young adults in Saudi Arabia during the COVID-19 pandemic. A high level of awareness of the likelihood of becoming infected was reported. The efficacy and safety of the COVID-19 vaccine were noted as barriers to vaccination. The benefits of vaccination included alleviating the anxiety of becoming infected. Incentivizing scientific research to demonstrate vaccine safety in the mid and long term should be encouraged by policy makers, including the MOH and Saudi CDC. Vaccine literacy programs should be designed according to the level of health, scientific, and general literacy of the high-priority groups. Strategies to reduce vaccination reluctance should be tailored to eliminate community-specific misconceptions about vaccination.

## Figures and Tables

**Table 1:** Demographics information.

	Number	Percent (%)
Age		
18–29	554	64.3
30–39	240	27.9
40–49	55	4.6
>50	13	1.5
Gender		
female	470	54.5
male	392	45.5
Education level		
High school and below	280	32.5
University education and above	582	67.5
Chronic conditions such as hypertension or diabetes		
Yes	52	6
No	810	94
Previous COVID-19 infection		
Yes	169	19.6
No	693	80.4
COVID-19 Vaccination history		
Yes	18	2.1
No	844	97.9
Vaccine registration through (Sehaty) app		
Yes	175	20.3
No	687	79.7

**Table 2:** Perceptions severity and likelihood of COVID-19 infection.

Statements	Strongly Disagree	Disagree	Neutral	Agree	Strongly Agree
	n (%)	n (%)	n (%)	n (%)	n (%)
Perceived Severity and Susceptibility					
COVID-19 infection is not serious so I don’t think I should take the vaccine	248 (29.5)	222 (26.4)	210 (25)	116 (13.8)	45 (5.4)
I have had COVID-19 infection and I think I’ve developed immunity against the virus	311 (36.9)	229 (27.2)	180 (21.4)	76 (9)	29 (3.4)
The likelihood of getting COVID-19 infection is low now, numbers are decreasing in Saudi Arabia (January, 2021)	304 (36.2)	230 (27.4)	172 (44.9)	102 (12.2)	30 (3.6)

**Table 3:** Reasons for hesitancy, perceived benefits, and causes of action.

Statements	Strongly Disagree	Disagree	Neutral	Agree	Strongly Agree
	n (%)	n (%)	n (%)	n (%)	n (%)
Perceived barrier (hesitancy)					
I’m not sure of eligibility and registration process	228 (27.4)	213 (25.6)	209 (25.1)	158 (18.9)	24 (2.9)
I’m not sure of the effectiveness of the vaccination	137 (16.4)	122 (14.6)	160 (19.1)	266 (31.8)	152 (18.1)
I believe that natural immunity is sufficient, and I don’t think I need to take the vaccine	206 (24.6)	211 (25.2)	175 (20.9)	146 (17.4)	99 (11.8)
I’ve heard on social media that the vaccine is not safe as it would contain the COVID-19 virus, so I’m worried about the side effects	224 (26.7)	147 (17.5)	190 (22.7)	195 (23.3)	82 (9.8)
Perceived benefits					
Taking the vaccine would make me less worried about catching COVID-19	164 (19.7)	164 (19.7)	178 (21.4)	238 (28.6)	87 (10.5)
Vaccination decreases my chance of getting COVID-19 or its complication	127 (15.2)	165 (19.8)	170 (20.4)	257 (30.8)	114 (17.2)
Vaccination would ease the precautionary measures including lock down, quarantine, and travel ban.	127 (15.2)	121 (14.5)	197 (23.6)	262 (31.4)	128 (15.3)
Causes of action					
I will only take the COVID-19 vaccine if I was given adequate information about it	164 (19.7)	164 (19.7)	178 (21.4)	238 (28.6)	87 (10.5)
I will only take the COVID-19 vaccine if the vaccine is taken by many in the public	127 (15.2)	165 (19.8)	170 (20.4)	257 (30.8)	114 (17.2)
I will only take the COVID-19 vaccine if it was made mandatory	127 (15.2)	121 (14.5)	197 (23.6)	262 (31.4)	128 (15.3)

## Data Availability

The data presented in this study are available on request from the corresponding author.
